# Deacetylforskolin ameliorates bleomycin-induced pulmonary fibrosis by suppressing inflammation and TGF-β1-induced epithelial–mesenchymal transition

**DOI:** 10.1007/s13659-026-00593-4

**Published:** 2026-04-13

**Authors:** Yan Zhong, Chuang Xiao, Yaping Liang, Peng Wang, Yun Long, Shuyi Li, Na Song, Wenbin Shang, Weimin Yang, Xuan Zhang

**Affiliations:** 1 School of Pharmaceutical Science and Yunnan Key Laboratory of Pharmacology for Natural Products/College of Modern Biomedical Industry, Kunming Medical University, Kunming, 650500 China; 2 The People’s Hospital of Dechang County, Liangshan Yi Autonomous Prefecture, Sichuan, 615500 China

**Keywords:** Pulmonary fibrosis, Deacetylforskolin, Inflammation, Epithelial–mesenchymal transition, MAPK signaling

## Abstract

Pulmonary fibrosis (PF) is a chronic, progressive and irreversible inflammatory disease with limited therapeutic methods in clinic. Deacetylforskolin (DFSK), derived from the plant *Coleus forskohlii*, is a potent adenylyl cyclase activator with potential anti-inflammatory activity. Herein, we attempted to investigate the therapeutic potential and mechanisms of DFSK against PF in bleomycin (BLM)-induced mouse models and TGF-β1-induced A549 cells. Our results showed that DFSK treatment alleviated lung injury and reduced inflammatory cytokines in a mouse model of BLM-induced acute lung inflammation, an early stage of PF. In a BLM-induced PF mouse model, DFSK attenuated pathological lung injury and collagen deposition, decreased pro-inflammatory cytokines (TNF-α, IL-1β) and profibrotic mediators (TGF-β1, CTGF, hydroxyproline). Upregulation of the epithelial marker E-cadherin and downregulation of the mesenchymal marker α-SMA were observed following DFSK treatment. Furthermore, DFSK significantly restored the pulmonary function of PF mice with decreased Te, f, Penh and increased RT, TV. Mechanistically, DFSK suppressed the phosphorylation of JNK and p38 MAPK, and inhibited TGF-β1-induced epithelial–mesenchymal transition (EMT) in A549 cells. Collectively, our findings demonstrate that DFSK is an effective therapeutic agent against PF by suppressing inflammation and EMT.

**Graphical Abstract:**

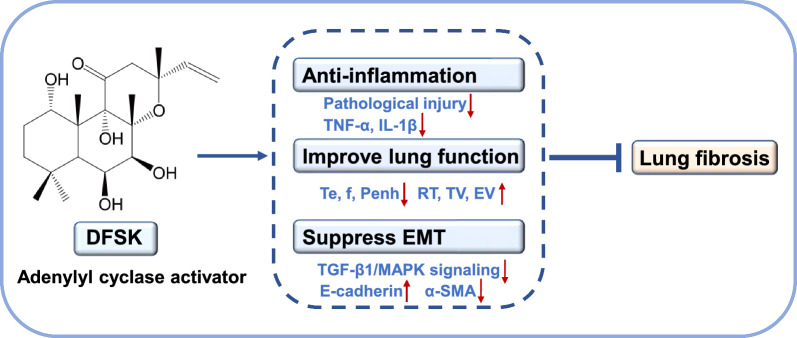

**Supplementary Information:**

The online version contains supplementary material available at 10.1007/s13659-026-00593-4.

## Introduction

Pulmonary fibrosis (PF) is a chronic and progressive inflammatory lung disease with high mortality [1, 2]. Environmental exposures such as cigarette smoke, viruses, silica, metal and wood dusts are considered as the risk factors of PF [1, 3]. PF is characterized by the remodeling of airways and alveoli, as well as the deposition of extracellular matrix [4]. The pathogenesis of PF is complex and involves multiple molecular pathways and cells including macrophages, fibroblasts, and epithelial cells [5]. The dysfunction of epithelial cells can promote the expression of transforming growth factor-β (TGF-β), which is a critical profibrotic mediator that drives epithelial–mesenchymal transition (EMT) and fibroblast-myofibroblast transition (FMT) [2, 4]. EMT is a dynamic process in which alveolar epithelial cells downregulate adhesion molecules like E-cadherin and begin expressing profibrotic markers such as α-SMA [6, 7]. This transition promotes the accumulation of myofibroblasts, which in turn secrete inflammatory cytokines and extracellular matrix (ECM) proteins, thereby driving the progression of PF [6].

The profibrotic effects of TGF-β are mediated by both the canonical Smad pathway and non-Smad pathways including Janus kinases (JAKs), Wnt, phosphatidylinositol-3-kinase (PI3K), Rho-associated kinases, and mitogen-activated protein kinase (MAPK) [5, 8]. The activation of MAPK (JNK, p38, and ERK) signaling pathways by TGF-β can promote cytoskeletal remodeling in EMT process and contribute to PF pathogenesis [6]. MAPK pathways can also be activated by reactive oxygen species to promote the proliferation of fibroblasts and excessive collagen deposition [6]. Furthermore, TGF-β induces the production of other profibrotic mediators such as fibroblast growth factor (FGF), connective tissue growth factor (CTGF), and platelet-derived growth factor (PDGF), which collectively participate in the pathological process of fibrosis [2, 9].

With the progress of research on the pathogenesis of PF in the past decades, the development of novel drugs against PF has been accelerated. Pirfenidone and nintedanib are representative drugs approved clinically for the treatment of PF, and they can prevent the decline of pulmonary function and slow down the disease progression [9]. However, these two drugs cannot improve the patients’ quality of life and are associated with tolerability problems [10]. Recently, nerandomilast (BI 1015550), a selective phosphodiesterase 4B (PDE4B) inhibitor that achieved the primary endpoint in phase III clinical trials [11, 12], has been approved by FDA for the treatment of PF. Nerandomilast treatment significantly attenuated the decline of forced vital capacity and was well-tolerated in patients [11, 12]. Roflumilast is another PDE4 inhibitor approved for the therapy of respiratory disease, which has been shown to reduce the risk of exacerbations in patients with chronic obstructive pulmonary disease (COPD) [13]. The pharmacological effects of PDE4 inhibition include anti-inflammation, bronchodilation and inhibition of airway remodeling, which are mediated by elevated intracellular cyclic adenosine monophosphate (cAMP) levels and its downstream signaling pathways [14]. Therefore, the modulation of intracellular cAMP levels represents a promising therapeutic strategy for obstructive pulmonary diseases including PF [15].

The homeostasis of cAMP is regulated by both adenylate cyclases (AC) which catalyze its formation and PDEs that mediate its hydrolysis [16]. Forskolin (FSK) and its derivatives are effective AC activators which can elevate the intracellular cAMP levels, and show therapeutic potential in the treatment of multiple diseases such as heart failure, neurodegenerative disorders, asthma, obesity, and glaucoma [17–19]. Notably, colforsin daropate (NKH477) is a water-soluble FSK derivative that selectively stimulates AC5 in the heart and is clinically used in Japan for acute heart failure [20, 21]. Isoforskolin (ISOF) and deacetylforskolin (DFSK) are natural products from *Coleus forskohlii* plants, and both are derivatives of FSK [22]. Previously, we have reported that ISOF significantly improved pulmonary function, attenuated inflammation, and promoted tracheal relaxation in COPD animal models [23, 24]. However, the therapeutic effects and mechanisms of AC activators on PF remain to be elucidated.

In this study, we aimed to investigate the anti-fibrotic effects of DFSK in a mouse model of PF and to explore its underlying mechanisms. Although the AC activation potency of DFSK is weaker than that of FSK or ISOF [17], DFSK exhibits superior equilibrium solubility in different solutions (Table S1), and DFSK possesses a favorable lipid-water partition coefficient (log P = 2.3), showing promising drug-like properties (Table S2). Furthermore, DFSK can stimulate the accumulation of cAMP in a concentration-dependent manner in HEK293 cells stably expressing AC isoforms (Fig. S1), indicating that it is a potent AC activator. In bleomycin (BLM)-induced mice models, the effects of DFSK on inflammation, PF, and lung function were assessed, and its effect on EMT was further investigated in vivo and in vitro. The results indicate that DFSK alleviates BLM-induced PF in mice by suppressing inflammation and EMT in which TGF-β1/MAPK signaling pathway may be involved. This finding underscores the promise of AC activators as potential anti-PF candidates.

## Materials and methods

### Reagents and antibodies

**Fig. 1:**
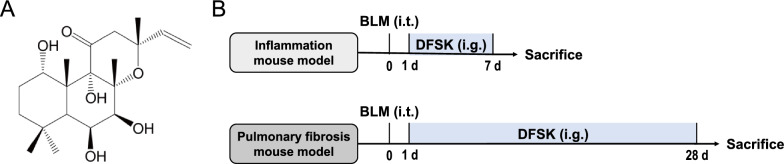
Experimental design. **A** The chemical structure of DFSK. **B** Experimental procedure for mouse models of inflammation or pulmonary fibrosis. Mice received a single intratracheal dose of BLM (5 mg/kg) under anesthesia, followed by intragastric administration of DFSK for 7 days (inflammation mouse model) or 28 days (pulmonary fibrosis mouse model) prior to endpoint analysis

DFSK (Fig. 1A, PubChem CID: 10044542) was obtained by deacetylation of ISOF [23], with a purity of over 99%. The mass spectrometry (TOF MS) spectroscopy of DFSK was shown in Figure S2. Pirfenidone (PFD) was purchased from Meilunbio (Dalian, China). Bleomycin hydrochloride (BLM) was obtained from Hanhui Pharmaceuticals (Hangzhou, China). SP600125 (JNK inhibitor), SB203580 (p38 MAPK inhibitor), and PD98059 (ERK1/2 signaling inhibitor) were purchased from MedChemExpress (New Jersey, USA). Hydroxyproline assay kit was from Nanjing Jiancheng Bioengineering Institute (Nanjing, China). Masson stain kit was obtained from Maxim Biotech (Fuzhou, China). The bicinchoninic acid (BCA) protein concentration assay kit was purchased from Beyotime Biotechnology (Shanghai, China). The cAMP ELISA kit was from Solarbio Technology (Beijing, China). Mouse TNF-α ELISA kit was from NeoBioscience Technology (Shenzhen, China). Mouse IL-1β ELISA kit was from Invitrogen (California, USA). Cell Counting Kit-8 (CCK-8), recombinant human TGF-β1 protein, mouse TGF-β1 ELISA kit, HRP-conjugated goat anti-rabbit IgG (H+L), rabbit TGF-β1 polyclonal antibody, and rabbit GAPDH polyclonal antibody were purchased from Proteintech (Chicago, USA). Mouse CTGF/CCN2 antibody was from R&D Systems (Minnesota, USA). Anti-alpha smooth muscle actin (α-SMA) antibody was from Abcam (Cambridge, UK). Rabbit E-cadherin polyclonal antibody, rabbit p38 MAPK antibody, SAPK/JNK antibody, p44/42 MAPK (ERK1/2) antibody, phospho-p38 MAPK (Thr180/Tyr182) antibody, phospho-SAPK/JNK (Thr183/Tyr185) antibody, phospho-p44/42 MAPK (ERK1/2) (Thr202/Tyr204) antibody were purchased from Cell Signaling Technology (Massachusetts, USA). All other reagents were of analytical grade and obtained commercially.

### Animals

Kunming mice (male, body weight 20 ± 2 g) were purchased from Department of Laboratory Animal Science, Kunming Medical University (Kunming, China). All animal care and experimental protocols were approved by the Animal Experimental Ethical Committee of Kunming Medical University (approval number: KMMU-2020111). After a 1-week acclimatization period, the mice were randomly assigned to the following six groups: sham, model, PFD (50 mg/kg), DFSK-L (5 mg/kg), DFSK-M (10 mg/kg) and DFSK-H (20 mg/kg) groups.

### BLM-induced inflammation model

Mice were subjected to intratracheal administration of BLM (5 mg/kg) on day 0 in model and treatment groups after anesthesia with 1% pentobarbital sodium (30 mg/kg). Mice in sham group were administered with an equal volume of 0.9% saline solution. Then, PFD and DFSK were administered intragastrically for 7 consecutive days in BLM-instilled mice. Mice were humanely euthanized on day 7 (Fig. 1B). Bronchoalveolar lavage fluid (BALF) and lung tissues of mice were subjected to further investigations.

### BLM-induced pulmonary fibrosis model

To establish the pulmonary fibrosis model, mice were intratracheally instilled with BLM (5 mg/kg) on day 0 and sacrificed on day 28. In treatment groups, PFD and DFSK were intragastrically administered once daily for 28 consecutive days. Mice in sham group and model group were orally administered with normal saline solution. On day 28, the pulmonary function was assessed in conscious mice, and then mice were euthanized (Fig. 1B). BALF and lung tissues of mice were collected for the following detections.

### Pulmonary function evaluation

Pulmonary function in conscious mice was detected by whole body plethysmography (EMKA technologies, Canada). Each mouse was placed in a chamber of the plethysmograph for 10–20 min for acclimatization. The respiratory waveforms were recorded for 5–10 min using the IOX software. The obtained respiratory parameters including expiratory time (Te), relaxation time (RT), respiratory frequency (f), enhanced pause (Penh), tidal volume (TV), expiratory volume (EV). The values for each parameter were averaged over a minimum of 10–15 consecutive stable breaths to obtain a representative value for each mouse.

### Lung histopathology

The lung tissues of each mouse were dissected and weighed to calculate the lung index (percentage of lung wet weight/ body weight). Subsequently, the tissues were immersed in 4% paraformaldehyde for fixation, followed by dehydration, paraffin embedding, and sectioning into 3–5 μm thick slices. The sections were stained with hematoxylin and eosin (H&E) for observation under a light microscope (Nikon Corporation, Japan). Pathological scoring of H&E-stained sections for inflammation and alveolar destruction was performed according to the reported method [25, 26]. The tissue sections were also stained with Masson trichrome to observe collagen deposition, and the fibrosis scores were determined as previously described [26, 27].

### Quantification of lung hydroxyproline

The hydroxyproline (Hyp) content in the lung tissues of mice were quantified using a commercial assay kit. Briefly, the left inferior lobe of lung tissue (40 mg) was hydrolyzed at 95 °C for 20 min. The hydrolysate was neutralized (pH 6.0–6.8), diluted to 10 mL, and purified via activated charcoal treatment and centrifugation. The Hyp concentration in the purified supernatant was then measured and calculated as specified by the manufacturer’s protocol.

### Enzyme-linked immunosorbent assay

Levels of TNF-α, IL-1β, and TGF-β1 in BALF were measured using commercial enzyme-linked immunosorbent assay (ELISA) kits. Briefly, BALF samples were centrifuged (2000 rpm, 10 min), and the obtained supernatants were detected with ELISA kits according to the manufacturer’s guidelines. The concentration of each sample was calculated through their respective standard curves.

### Cell culture

The human A549 alveolar epithelial cell line (Cat No. CL-0190, Procell system, Wuhan, China) were cultured in complete DMEM/F12 medium, which was composed of a 1:1 mixture of DMEM and Ham’s F-12 medium, supplemented with 10% (v/v) fetal bovine serum (FBS) and 1% (v/v) penicillin–streptomycin. The cells were maintained under standard culture conditions (37°C, 5% CO_2_ in a humidified incubator). The cell culture medium was replaced every 2–3 days, and cells were treated with 0.25% trypsin upon reaching 80–90% confluence. Cells were routinely harvested at the logarithmic growth stage for all experimental procedures.

### Cell viability detection

Cell viability was detected though the CCK-8 assay. Cells were seeded at a density of 5 × 10^4^ cells/mL in 96-well plates, with 100 μL cell suspension adding to each well. Following 24 h of incubation, cells were treated with PBS buffer, TGF-β1 (10 ng/mL), and gradient concentrations of DFSK (2.5, 5, 10, 25, 50, 100, 200 µM). After incubating for 48 h, cell viability was determined by adding 10 μL of CCK-8 reagent per well, incubating for 2 h at 37 °C, and measuring the absorbance at 450 nm with a Multiskan GO microplate reader (ThermoFisher, USA).

### TGF-β1 induced EMT cell model

A549 cells were induced to undergo EMT by treatment with TGF-β1 (10 ng/mL) [28]. Cells were divided into five experimental groups: control group, TGF-β1-induced EMT group, and TGF-β1 with 50, 100, or 200 µM DFSK groups. A549 cells were seeded at a density of 2 × 10^5^ cells per well in 6-well plates and cultured for 24 h. Cells were preincubated with different concentrations of DFSK for 1 h, then TGF-β1 were added to the cell culture. After incubating for 48 h, cell morphology was assessed under phase-contrast microscopy (Olympus, Japan), and cells were harvested for subsequent experiments. To investigate the role of MAPK signaling in DFSK’s effects, TGF-β1-induced A549 cells were pretreated for 1 h with MAPK pathway inhibitors (10 μM SP600125, SB203580, or PD98059) as positive controls. For intracellular cAMP detection, cells were harvested after TGF-β1 or DFSK stimulation for 30 min, then cells were lysed according to the method described previously [29]. The cAMP concentration in the lysates (200 µL) was quantified using a commercial ELISA kit.

### Western blot analysis

Following homogenization of lung tissue or cell samples in RIPA buffer, protein concentrations were quantified by the BCA assay kit. Subsequently, equal amounts of protein (30 μg) were electrophoresed on 12% SDS-PAGE gels and transferred to polyvinylidene difluoride membranes. The membranes were blocked using a blocking buffer (Beyotime, China) for 2 h at room temperature. Then, the membranes were incubated with the following primary antibodies at 4 °C overnight: CTGF, TGF-β1, E-cadherin, α-SMA, p-p38, p38, p-JNK, JNK, p-ERK, ERK, and GAPDH. Following three washes, the membranes were incubated with HRP‐conjugated secondary antibodies for 2 h at room temperature. The membranes were then detected with a supersensitive ECL chemiluminescence detection reagent (Bio-Rad Laboratories, USA), and the signals of protein bands were acquired by an Amersham Imager 600 ultrasensitive multi-function imager (Cytiva, USA). The relative expression levels of proteins were quantified using the ImageJ software (National Institute of Health, USA).

### Statistical analysis

Data were expressed as the mean ± SEM from a minimum of three independent experiments. Statistical analyses were performed by GraphPad Prism 9 (GraphPad Software, USA). Differences among multiple groups were assessed using one-way analysis of variance (ANOVA), followed by Fisher’s least significant difference (LSD) test. If the assumption of equal variances was violated, Brown-Forsythe and Welch ANOVA tests were used. For data that deviated from a normal distribution, the non-parametric Kruskal–Wallis test was applied. Statistical significance was set at *P* < 0.05.

## Results

### DFSK ameliorated BLM-induced lung inflammation in mice

**Fig. 2:**
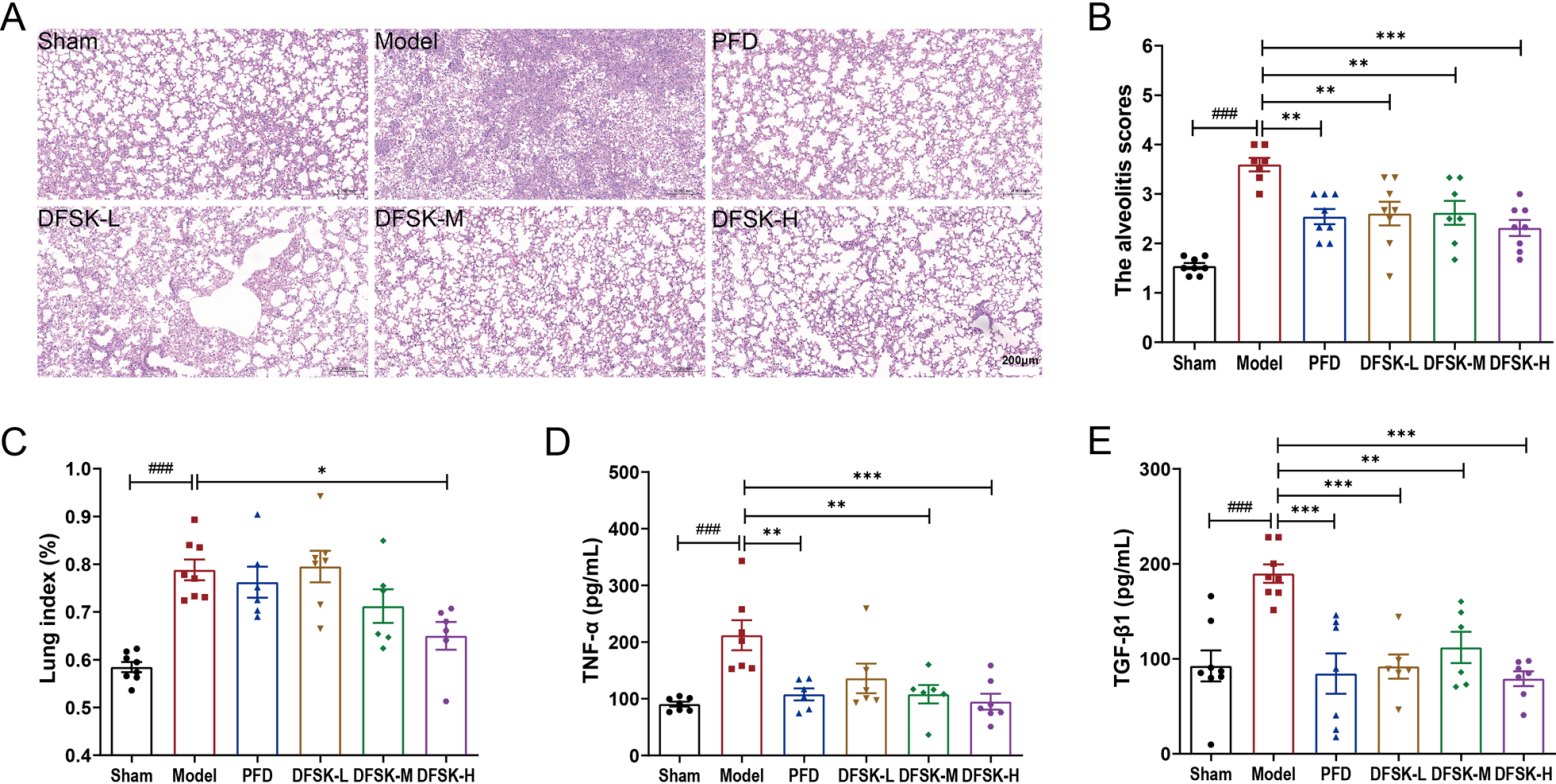
DFSK ameliorated BLM-induced pulmonary inflammation in mice. **A** Representative H&E-stained lung sections (scale bar, 200 μm). **B** The alveolitis scores of mice. **C** Lung index of mice. **D** TNF-α levels in BALF of mice. **E** TGF-β1 levels in BALF of mice. Data are shown as the mean ± SEM (n = 6–8). ^###^*P* < 0.001 versus sham group, ^*^P < 0.05, ^**^P < 0.01, ^***^P < 0.001 versus model group

To investigate the therapeutic potential of DFSK in the early inflammatory phase of pulmonary fibrosis, we evaluated its effects in mice with BLM-induced acute lung injury. Mice subjected to intratracheal BLM instillation were treated with DFSK (5, 10, 20 mg/kg) via intragastric administration for 7 days. Histopathological examination by H&E staining showed that BLM triggered a prominent influx of inflammatory cells into the interstitium and airspaces, along with prominent alveolar septal thickening and collapsed alveolar cavities. These pathological alterations were notably ameliorated by DFSK or PFD treatment (Fig. 2A). Meanwhile, the histopathological alveolitis scores were significantly elevated in the model group but reduced in the DFSK or PFD treated group (Fig. 2B). The lung index, calculated as the ratio of lung wet weight to body weight, was markedly increased in model mice, indicating the development of pulmonary edema and inflammation. DFSK treatment at 20 mg/kg significantly decreased the lung index of mice (Fig. 2C). Furthermore, ELISA analysis showed BLM-induced elevations of inflammatory cytokines (TNF-α, TGF-β1) in BALF were significantly suppressed by DFSK or PFD treatment (Fig. 2D-E). Thus, these results demonstrate that DFSK effectively ameliorates BLM-induced acute lung inflammation in mice.

### DFSK attenuated BLM-induced PF in mice

**Fig. 3:**
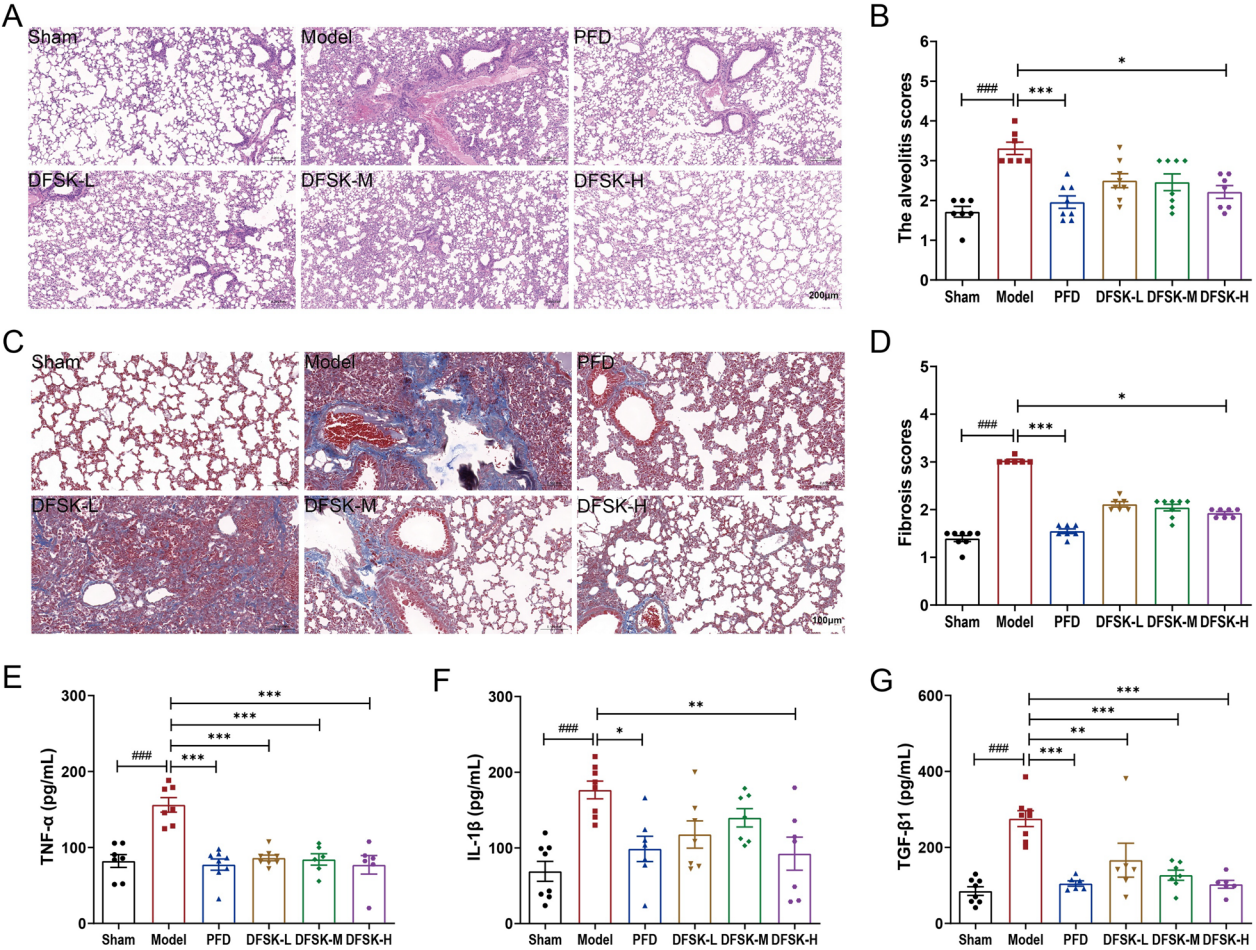
DFSK attenuated BLM-induced pulmonary fibrosis in mice. **A** Representative H&E-stained lung sections (scale bar, 200 μm). **B** The alveolitis scores of mice in each group. **C** Masson’s trichrome staining of lung tissues (scale bar, 100 μm). **D** The fibrosis scores of mice in each group. **E** TNF-α levels in BALF of mice. **F** IL-1β levels in BALF of mice. **G** TGF-β1 levels in BALF of mice. Data are shown as the mean ± SEM (n = 6–8). ^###^*P* < 0.001 versus sham group, ^*^P < 0.05, ^**^P < 0.01, ^***^P < 0.001 versus model group

We further evaluated the effect of DFSK on BLM-induced pulmonary fibrosis in mice. Following the induction of pulmonary fibrosis via intratracheal instillation of BLM, mice were treated with DFSK for 4 weeks. Lung tissue pathology in mice was evaluated using H&E and Masson’s trichrome staining. The results showed that BLM-induced model mice displayed extensive inflammatory cell infiltration, alveolar hemorrhage, thickened alveolar walls and widened septa, and increased collagen deposition in the pulmonary interstitium (Fig. 3A, C). Treatment with DFSK or PFD markedly alleviated these pathological alterations and suppressed pulmonary interstitial collagen deposition (Fig. 3A, C). In comparison with the model group, the histopathological scores for alveolitis and fibrosis were also significantly decreased in DFSK (20 mg/kg) or PFD treated group (Fig. 3B, D). Moreover, DFSK and PFD significantly suppressed the BLM-induced elevation of pro-inflammatory and profibrotic mediators (TNF-α, IL-1β, and TGF-β1) in BALF (Fig. 3E–G). Collectively, a higher dose of DFSK exhibited comparable efficacy to PFD in this study, indicating its potent therapeutic effect against BLM-induced PF in mice.

### DFSK improved lung function in BLM-induced PF mice

**Fig. 4:**
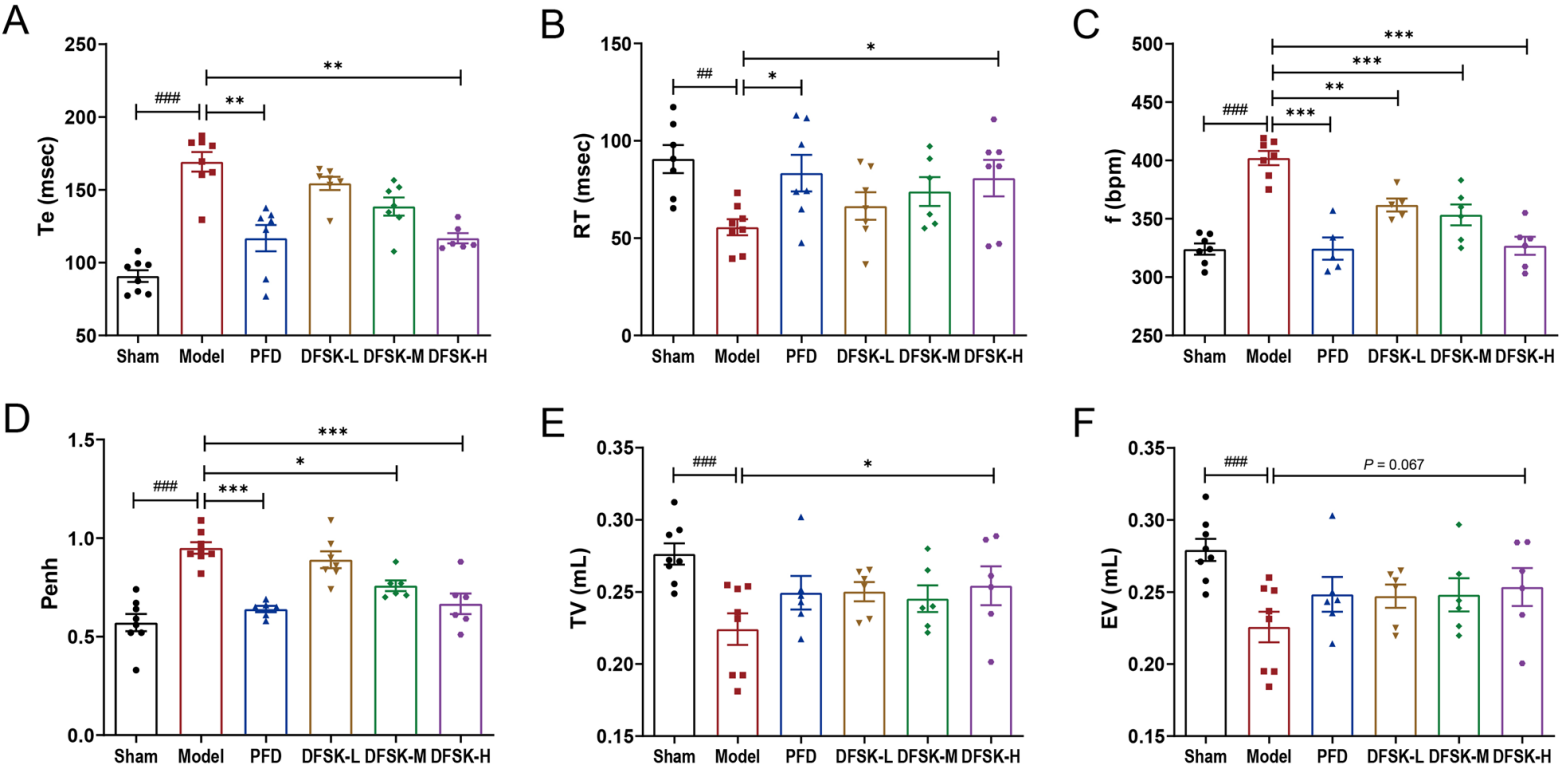
DFSK improved lung function in BLM-induced PF mice. **A** Expiratory time (Te) in mice of each group. **B** Relaxation time (RT) in mice of each group. **C** Respiratory frequency (f) in mice of each group. **D** Enhanced pause (Penh) in mice of each group. **E** Tidal volume (TV) in mice of each group. **F** Expiratory volume (EV) in mice of each group. Data are shown as the mean ± SEM (n = 6–8). ^##^*P* < 0.01, ^###^*P* < 0.001 versus sham group, ^*^P < 0.05, ^**^P < 0.01, ^***^P < 0.001 versus model group

PF is characterized by a progressive loss of lung function, which is central to its poor prognosis [30]. At the endpoint of the experiment, lung function parameters were measured in conscious mice by whole body plethysmography. Te represents the duration of expiration correlated with lung compliance, f is the respiratory rate and often increases compensatively to maintain ventilation, and Penh is an index of airway resistance that associated with bronchoconstriction or airway remodeling. RT reflects the balance between lung elastance and airway resistance, while TV (volume of normal inhalation and exhalation) and EV (expiratory volume) represent the pulmonary ventilation function. Compared with the sham group, mice in model group exhibited elevated Te, f, and Penh, alongside significantly reduced RT, TV, and EV. The results indicated that tracheal instillation of BLM resulted in increased airway resistance, and decreased lung compliance in mice (Fig. 4A–F). Treatment with DFSK or PFD ameliorated the BLM-induced impairment of lung function to varying degrees (Fig. 4A–F). Notably, a higher dose of DFSK (20 mg/kg) showed significant improvements in lung function parameters (decreased Te, f, Penh and increased RT, TV), which were comparable to the positive control PFD. These results suggest that DFSK can restore lung function in BLM-induced PF mice.

### DFSK suppressed BLM-induced EMT in mice

**Fig. 5:**
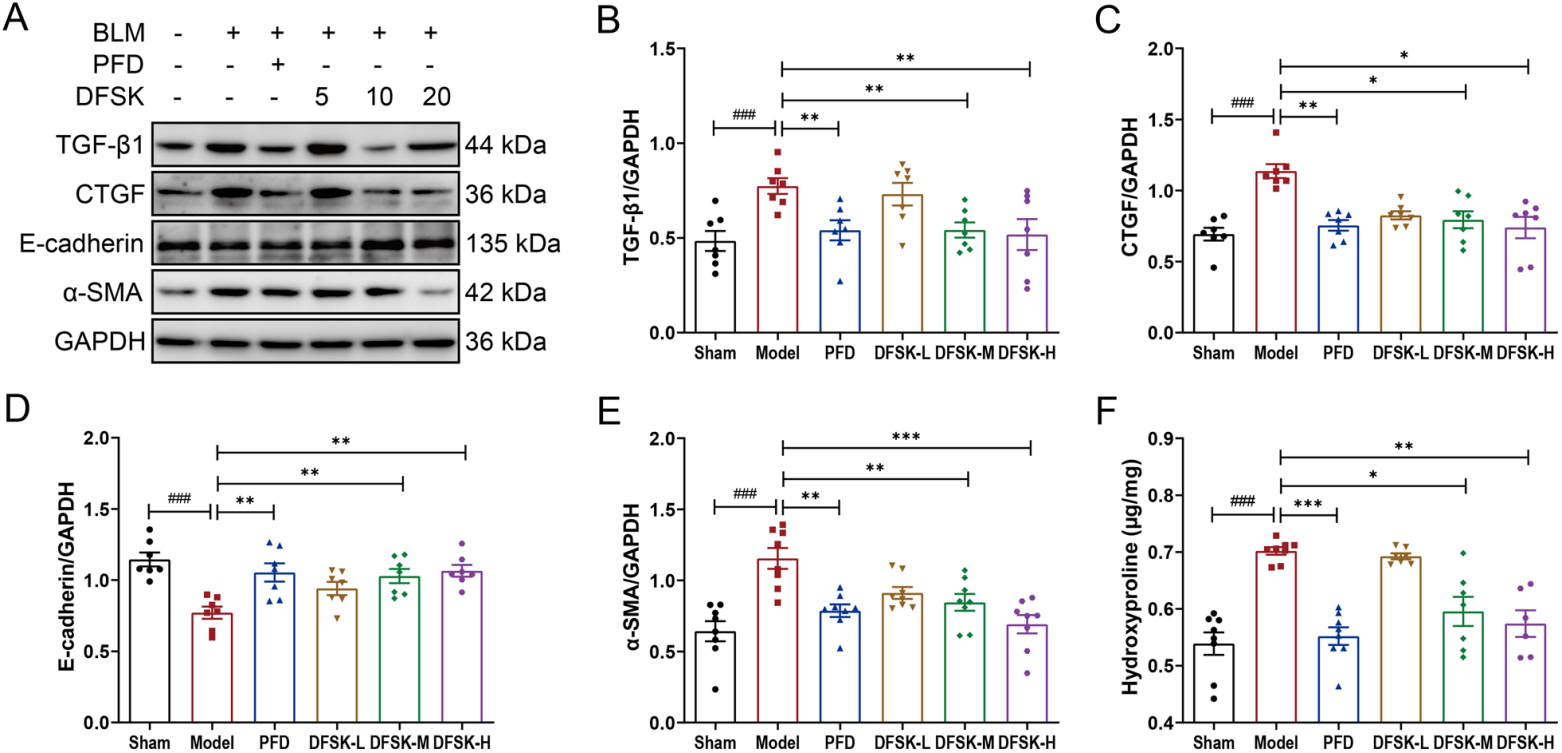
DFSK suppressed BLM-induced EMT in mice. **A** The representative western blot images of TGF-β1, CTGF, E-cadherin, and α-SMA protein expression levels in mouse lung tissues. **B**–**E** The protein levels of TGF-β1, CTGF, E-cadherin, and α-SMA were normalized to GAPDH and quantified by densitometry. **F** The hydroxyproline content in the lung tissues of mice. Data are shown as the mean ± SEM (n = 6–8). ^###^*P* < 0.001 versus sham group, ^*^P < 0.05, ^**^P < 0.01, ^***^P < 0.001 versus model group

TGF-β1 and CTGF are pivotal drivers of lung fibrosis through multiple pathological processes including EMT, which is histologically defined by the loss of epithelial markers such as E-cadherin and the concurrent acquisition of mesenchymal markers like α-SMA [4, 31]. To evaluate the impact of DFSK on EMT in pulmonary fibrosis mice, the protein levels of TGF-β1, CTGF, E-cadherin, and α-SMA in mouse lung tissues were analyzed by western blot (Fig. 5A–E). The results confirmed that BLM significantly promoted TGF-β1, CTGF, and α-SMA expression, while downregulated E-cadherin in mice. Both DFSK (10, 20 mg/kg) and PFD treatment reversed these protein expression changes in model mice, indicating that higher doses of DFSK could effectively suppress BLM-induced EMT. In addition, the Hyp content in the lung tissues, which serves as a key indicator of collagen accumulation, was significantly elevated in BLM-induced mice (Fig. 5F). Treatment with DFSK (10, 20 mg/kg) or PFD significantly reduced the Hyp content, further confirmed their efficacy in inhibiting collagen deposition during fibrosis progression.

### DFSK reduced TGF-β1-induced EMT in A549 cells

**Fig. 6:**
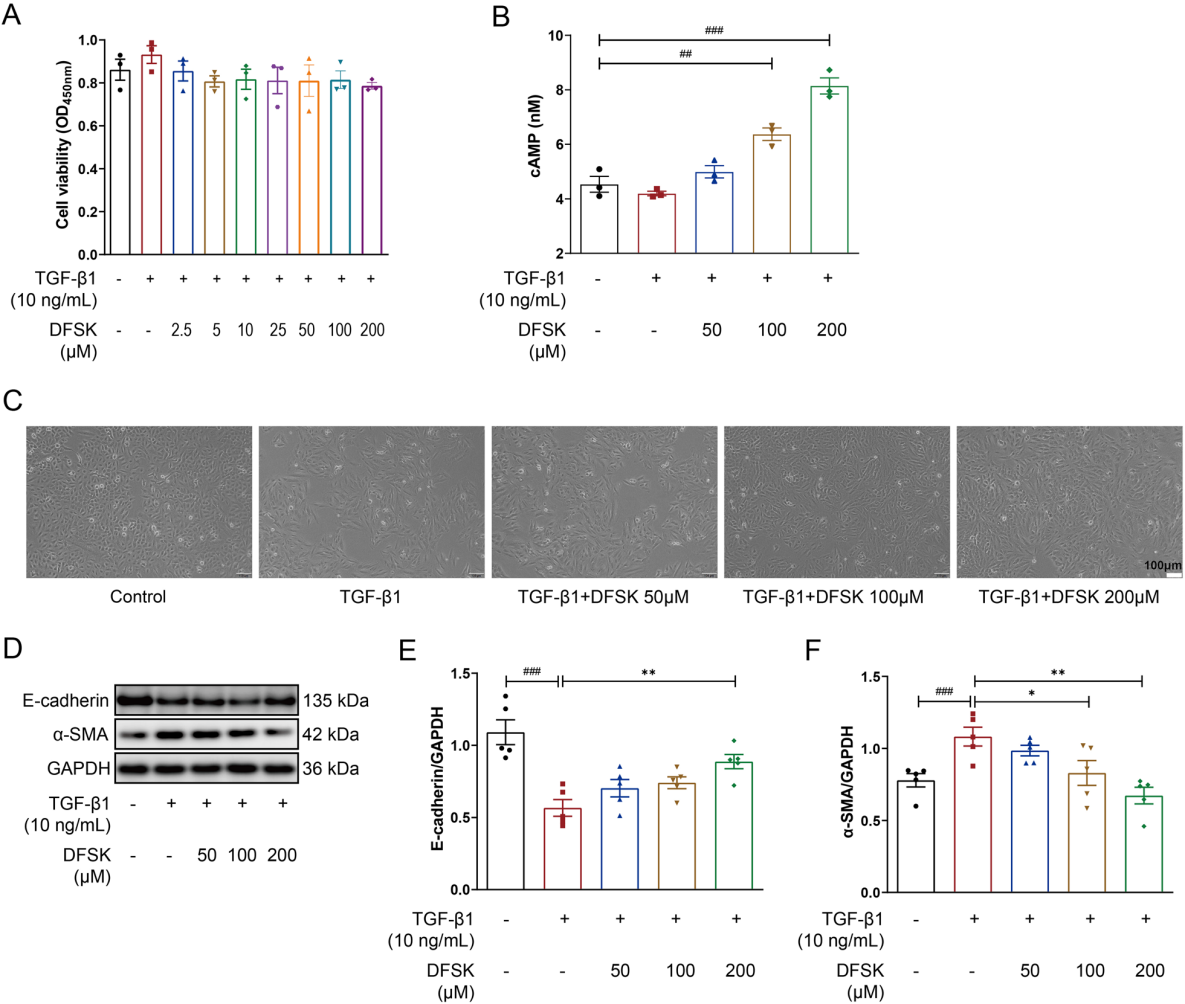
DFSK reduced TGF-β1-induced EMT in A549 cells. **A** Effects of TGF-β1 and DFSK (2.5 to 200 μM) on A549 cell viability detected by CCK-8 assay. **B** The intracellular cAMP levels in A549 cells. **C** The morphology of A549 cells after TGF-β1 or DFSK treatment (original magnification: 100×). **D** The representative western blot images of E-cadherin and α-SMA protein expression levels in A549 cells. **E**, **F** The protein levels of E-cadherin and α-SMA were normalized to GAPDH and quantified by densitometry. Data are shown as the mean ± SEM (n = 3 or 5). ^##^*P* < 0.01, ^###^*P* < 0.001 versus control group, ^*^P < 0.05, ^**^P < 0.01 versus TGF-β1-treated group

To elucidate the anti-fibrotic mechanisms of DFSK, we employed an in vitro EMT model using A549 alveolar epithelial cells. A549 cells were stimulated with 10 ng/mL TGF-β1 for 48 h. First, the effect of TGF-β1 and DFSK on cell viability was assessed by the CCK-8 assay. The results showed that TGF-β1 could slightly promote A549 cell proliferation, while DFSK (2.5 to 200 μM) exerted no significant impact on cell viability in the presence of TGF-β1 (Fig. 6A). Then, DFSK at concentrations of 50, 100, and 200 μM were used in subsequent experiments. The intracellular cAMP levels were detected after TGF-β1 or DFSK stimulation, and the results showed that DFSK could dose-dependently elevate cAMP levels in A549 cells (Fig. 6B). Morphological assessment by phase-contrast microscopy showed that A549 cells in the control group exhibited the classic epithelial phenotype with polygonal and cobblestone-like appearance (Fig. 6C). In contrast, TGF-β1 stimulation induced a marked transition toward a mesenchymal phenotype with elongated spindle-like shape and disrupted cell contacts. Notably, DFSK treatment attenuated these TGF-β1-induced morphological changes, and a more epithelial-like morphology was observed at higher concentrations of DFSK (Fig. 6C). Consistent with the morphological observations, TGF-β1 stimulation induced an EMT phenotype of A549 cells, characterized by a marked downregulation of E-cadherin and an upregulation of α-SMA (Fig. 6D–F). DFSK treatment suppressed TGF-β1-induced EMT in A549 cells in a dose-dependent manner, where 200 μM DFSK upregulated E-cadherin and downregulated α-SMA expression (Fig. 6D–F). The results were consistent with the effect of DFSK on BLM-induced mice model, suggesting that DFSK could attenuate pulmonary fibrosis though inhibiting EMT.

### DFSK suppressed MAPK signaling in TGF-β1-induced A549 cells

**Fig. 7:**
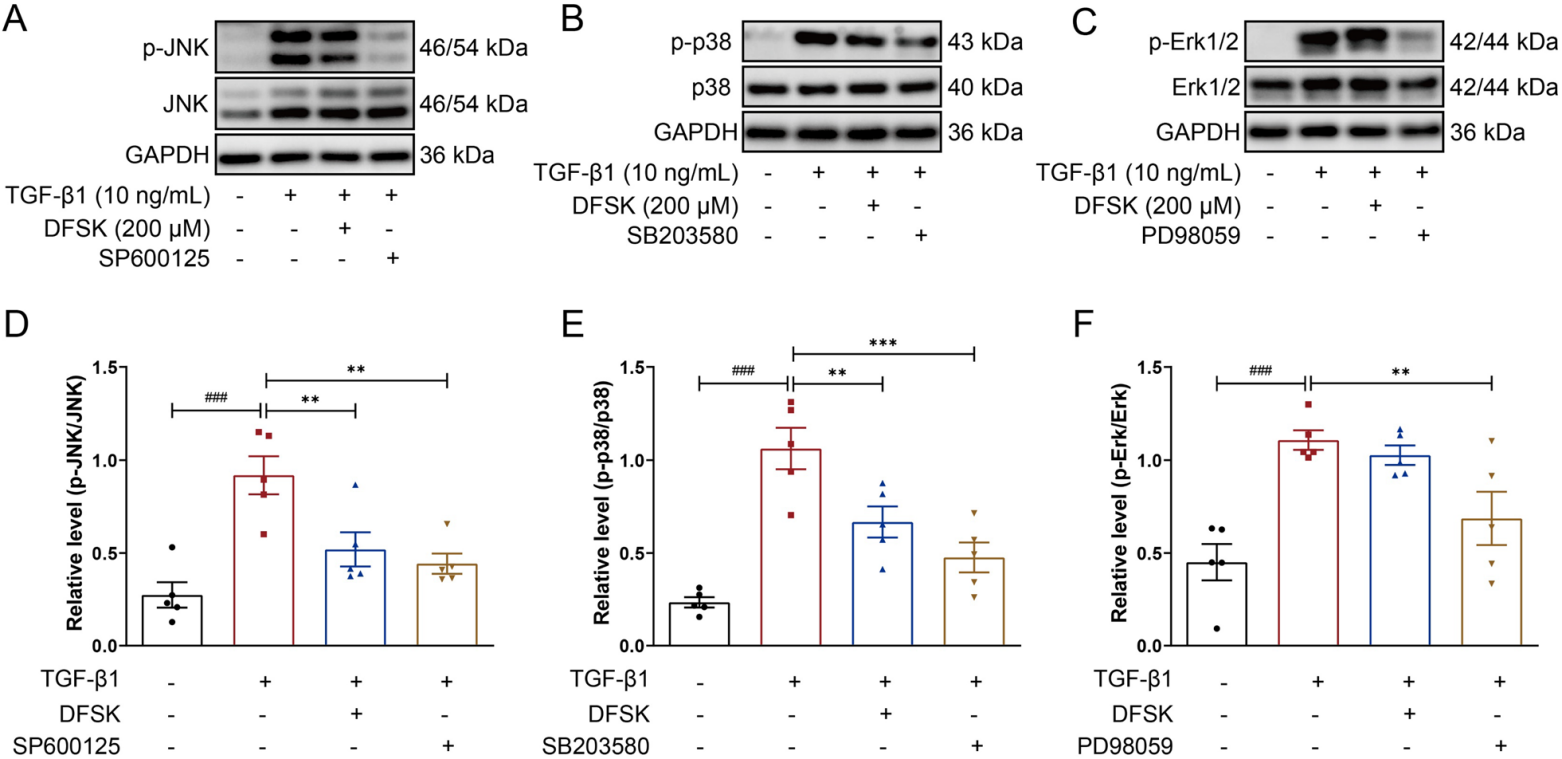
DFSK suppressed MAPK signaling in TGF-β1-induced A549 cells. **A**-**C** The representative western blot images of p-JNK, JNK, p-p38, p38, p-ERK, and ERK protein expression levels in A549 cells. **D** The relative density of p-JNK to JNK in each group. **E** The relative density of p-p38 to p38 in each group. **F** The relative density of p-ERK to ERK in each group. Data are shown as the mean ± SEM (n = 5). ^###^*P* < 0.001 versus control group, ^*^P < 0.05, ^**^P < 0.01 versus TGF-β1-treated group

TGF-β1-induced activation of MAPK signaling is one of the non-canonical pathways in fibrosis [8]. MAPKs are mainly composed of p38, JNK, and ERK, which play important roles in EMT and pulmonary fibrosis progression [6]. Given that DFSK is an AC activator which can elevate intracellular cAMP level and regulate downstream pathways including MAPK [32], we further explored the effect of DFSK on MAPK signaling pathway in TGF-β1-induced A549 cells. The specific inhibitors of p38, JNK, or ERK pathway were used as positive controls. The results showed that the phosphorylation of JNK, p38, and ERK were markedly enhanced in TGF-β1-induced A549 cells (Fig. 7A–F). As expected, SP600125, SB203580, and PD98059 effectively inhibited the phosphorylation of JNK, p38, and ERK respectively (Fig. 7A–F). DFSK (200 μM) significantly inhibited the phosphorylation of JNK and p38, but had little effect on the phosphorylation of ERK (Fig. 7A–F). Thus, these results indicate that DFSK might inhibit TGF-β1-induced EMT in A549 cells though suppressing JNK and p38 MAPK signaling pathways.

## Discussion

Pulmonary fibrosis is a chronic, progressive, and fatal interstitial lung disease characterized by aberrant proliferation of fibroblasts and excessive deposition of ECM proteins, leading to the destruction of normal lung architecture and progressively deteriorating respiratory function [1, 5]. Current treatment options for pulmonary fibrosis remain limited. In this study, we report that DFSK, an analog of diterpene forskolin, can effectively ameliorate BLM-induced inflammation and pulmonary fibrosis, and improve pulmonary function in mice. Further studies demonstrate that DFSK can suppress EMT process both in vivo and in vitro, which might be mediated through the TGF-β1/MAPK signaling pathway. Our results underscore the therapeutic potential of AC activators, including DFSK, as promising candidates for PF.

Despite considerable efforts in anti-PF drug development during the last two decades, the majority of clinical trials have been unsuccessful [2]. Pirfenidone and nintedanib have received approval for the management of PF over 10 years. Although they showed efficacy in slowing the progression of FVC decline and lowering mortality risk, they are not curative and are often limited by tolerability issues [2, 10]. A novel selective PDE4B inhibitor, nerandomilast, is a recently FDA-approved drug for the treatment of PF. This drug demonstrates combined anti-inflammatory and anti-fibrotic efficacy with improved tolerability over existing therapies [33]. As a PDE4 inhibitor, roflumilast has been demonstrated to alleviate BLM-induced lung injury and fibrosis in mice [34]. The pharmacological effects of PDE4 inhibitors are mediated by intracellular cAMP signaling, which is also regulated by AC [16]. We therefore hypothesize that AC activators may exert similar protective effects against PF. While FSK and its analogs are established AC activators, DFSK represents a promising candidate due to its superior physicochemical characteristics compared to FSK or ISOF (Tables S1, 2).

Inflammatory response plays a critical role in the progression of PF, particularly during acute exacerbation of the disease [5, 35]. In a BLM-induced inflammatory mouse model, DFSK treatment significantly attenuated the histopathological damage and reduced the alveolitis score, lung index, as well as levels of inflammatory cytokines (Fig. 2). The results indicate that DFSK could ameliorate BLM-induced acute lung inflammation in mice. This finding, together with our previous reports that ISOF could attenuate LPS-induced inflammation and acute lung injury in vivo and in vitro [36–38], further supports the broad anti-inflammatory potential of AC activators. In an established model of BLM-induced pulmonary fibrosis, DFSK alleviated pulmonary interstitial collagen deposition with decreased alveolitis and fibrosis scores, reduced pro-inflammatory and profibrotic cytokines in BALF, and improved lung function in mice (Figs. 3, 4). Moreover, DFSK suppressed BLM-induced EMT, as indicated by reduced TGF-β1, CTGF, and α-SMA expression and increased E-cadherin expression (Fig. 5). A post-hoc power analysis was performed on data from the mouse models using the G*Power software, and the calculated statistical power exceeded 0.88. Generally, post-hoc power analysis of small samples can be unstable and tend to be optimistic. The high post-hoc power value reflects that the observed effects were substantial relative to the variability in our data, suggesting that the modest sample size was statistically adequate for the primary aims of this study. Collectively, these results demonstrate that DFSK is an effective therapeutic agent against PF through inhibition of inflammation and EMT. The anti-fibrotic effects of other cAMP-elevating agents have also been reported. The PDE4 inhibitor roflumilast could suppress the expression of TNF-α, TGF-β1, CTGF, collagen, and Muc5ac, showing both preventive and therapeutic effects in BLM-induced lung fibrosis mouse and rat models [34]. Furthermore, FSK reduced oxidative stress and inflammation cytokines, inhibited collagen deposition as well as α-SMA expression in a CCl_4_-induced liver fibrosis rat model [39]. Therefore, the cAMP-elevating agents demonstrate multiple anti-fibrotic effects in experimental animal models. It should be noted that only male mice were used in this study in order to avoid the effects of physiological variability especially variable hormone levels in female mice. However, for a comprehensive efficacy evaluation in drug development, future studies employing both male and female animals are required to confirm the effectiveness of DFSK against PF.

TGF-β1 belongs to the TGF-β superfamily, and is a pivotal mediator in EMT and the pathogenesis of PF [40]. In TGF-β1-induced A549 cell model, DFSK dose-dependently downregulated α-SMA expression and upregulated E-cadherin expression, thereby inhibiting the EMT phenotype of model cells (Fig. 6). Mechanistically, DFSK inhibited the phosphorylation of JNK and p38 MAPK (Fig. 7), thus blocking the non-canonical TGF-β1/MAPK pathway in fibrosis. The crosstalk of cAMP and MAPK signaling has been established in different cell types [32, 41–43]. By elevating intracellular cAMP, FSK inhibited TGF-β-induced expression of profibrotic mediators (CTGF, collagen, TIMP-1, PAI-1) and prevented the transactivation of Smad-dependent gene in human dermal fibroblasts, while without inhibiting the phosphorylation or nuclear translocation of Smad protein [42]. In TGF-β1-induced MDCK cells, FSK had no effect on the phosphorylation of Smad proteins or p38 MAPK, whereas it significantly inhibited the activation of ERK [43]. A recent study identified MAPK phosphatase 1 (MKP1) as a critical antifibrotic protein in PF resolution, showing that MKP1 could be upregulated by FSK and subsequently promoted p38 dephosphorylation in lung myofibroblasts [44]. Thus, the anti-fibrotic effects of cAMP signaling involve multiple cell types and avenues. Further studies are required to elucidate the specific effect of DFSK on fibroblast-myofibroblast transition in PF.

The results in this study showed that DFSK could inhibit the phosphorylation of JNK and p38 but has no effect on ERK. This may be related to the different functions and upstream signals of JNK/p38 and ERK. ERK pathway (Ras/Raf/MEK/ERK) is mainly activated by growth factors to support cell proliferation and survival, while the activation of JNK and p38 is associated with cell apoptosis [45]. Moreover, increased intracellular cAMP can modulate MAPK signaling through PKA and EPAC proteins. PKA can inhibit the activation of ERK signaling in multiple cell types, while EPAC may enhance ERK signaling [46, 47]. A previous study showed that complement protein C1q, a cAMP-elevating agent that inhibit cell proliferation at sites of inflammation, increased cAMP-dependent protein kinase I level with no effect on ERK activation in human fibroblasts [48]. The inhibition of endoplasmic reticulum stress can alleviate NiCl_2_-induced EMT in A549 cells, which might through suppressing Smad2/3 and p38 MAPK pathways, but not ERK and JNK MAPK pathways [49]. Therefore, the spatial and temporal dynamics of intracellular cAMP or MAPK signaling may contribute to the regulation of specific cellular functions by extracellular factors [15, 50].

The development of cAMP-elevating agents targeting specific tissues represents a promising strategy for anti-fibrotic therapy [51]. Nerandomilast, a selective PDE4B inhibitor recently approved for the treatment of PF, elevates intracellular cAMP levels to exert anti-inflammatory and anti-fibrotic effects [33]. Compared to current therapies, nerandomilast can prevent disease progression with fewer adverse effects, highlighting the promise of cAMP-mediated anti-fibrotic effects [52]. Previous studies have reported the anti-fibrotic effects of FSK in liver fibrosis and cholestatic liver disease models [39, 53]. Here, we demonstrate for the first time that DFSK is an effective therapeutic agent against PF by suppressing inflammation and EMT, supporting AC as a promising therapeutic target for PF. Mammals express nine transmembrane AC isoforms with distinct tissue distributions, and this diversity poses a major challenge for developing selective AC activators to achieve tissue-specific targeting [19, 54]. Colforsin daropate (NKH477) is a water-soluble FSK derivative with enhanced selectivity for the AC5 isoform, and it is clinically used in Japan against acute heart failure [20, 21]. Our previous research showed that ISOF has potent tracheal relaxant effects, which are stronger than that of PDE4 inhibitors [23]. Therefore, in addition to improved physicochemical properties, the tracheal relaxant effect of AC activators is also a potential advantage that may contribute to the anti-PF efficacy of DFSK. A previous pharmacokinetic study showed that ISOF can be absorbed rapidly and has good bioavailability after oral administration in guinea pigs [55]. Given that DFSK is not a selective AC isoform activator [17], we have not evaluated its pharmacokinetic and pharmacodynamic properties, which is a limitation of this study. The lack of selectivity for AC isoforms may be a potential risk for clinical applications of AC activators [18]. Thus, it is still challenging for the clinical translation of DFSK despite its favorable physicochemical properties and efficacy. Therefore, DFSK might be a suitable lead compound, and structural optimization remains crucial for developing selective AC isoform activators as novel therapeutics against lung fibrosis in the future.

## Conclusion

Collectively, our work demonstrates that DFSK alleviates BLM-induced pulmonary inflammation, fibrosis and lung function decline in mice, and also inhibits TGF-β1-induced EMT in vitro through MAPK signaling pathway. These findings indicate that DFSK is a promising therapeutic agent for PF, and underscore the potential of AC activation as a viable anti-fibrotic strategy.

## Supplementary Information

Supplementary Material 1.

## Data Availability

All data generated in this study are available within the article or Supplementary Material. Additional inquiries should be directed to the corresponding authors.
