# Supplementary material for: Deacetylforskolin ameliorates bleomycin-induced pulmonary fibrosis by suppressing inflammation and TGF-β1-induced epithelial–mesenchymal transition
Source: Nat Prod Bioprospect. 2026 Apr 13;16(1):52. doi: 10.1007/s13659-026-00593-4 (PMC13070883; doi:10.1007/s13659-026-00593-4)
Supplement: Supplementary file 1 — Supplementary Material 1. [file 13659_2026_593_MOESM1_ESM.docx]

Supplementary Material:

Deacetylforskolin ameliorates bleomycin-induced pulmonary fibrosis by suppressing inflammation and TGF-β1-induced epithelial-mesenchymal transition

**Yan Zhong^1, 2†^, Chuang Xiao^1†^, Yaping Liang^1^, Peng Wang^1^, Yun Long^1^,** **Shuyi Li^1^, Na Song^1^, Wenbin Shang^1*^, Weimin Yang^1*^, Xuan** **Zhang^1*^**

^1^ School of Pharmaceutical Science and Yunnan Key Laboratory of Pharmacology for Natural Products / College of Modern Biomedical Industry, Kunming Medical University, Kunming, 650500, China

^2^ The People’s Hospital of Dechang County, Liangshan Yi Autonomous Prefecture, Sichuan, 615500, China

^†^These authors have contributed equally to this work.

**^*^Correspondence:** Wenbin Shang (shangwenbin@kmmu.edu.cn); Weimin Yang (ywmbessie@yeah.net); Xuan Zhang (zhangxuan@kmmu.edu.cn).

**Supplementary Figure:**

**Fig. S1** Concentration-response curve of DFSK stimulating cAMP production in HEK293 cells overexpressing hAC1 or hAC2. The AC activation potency of DFSK was detected by a FRET-based assay in HEK293 cells overexpressing hAC1 or hAC2 as previously described (Xiao C. *Phytomedicine*. 2021, 91:153701). The calculated EC_50_ values of DFSK were 11.76 ± 1.14 μM and 10.65 ± 1.07 μM, respectively.


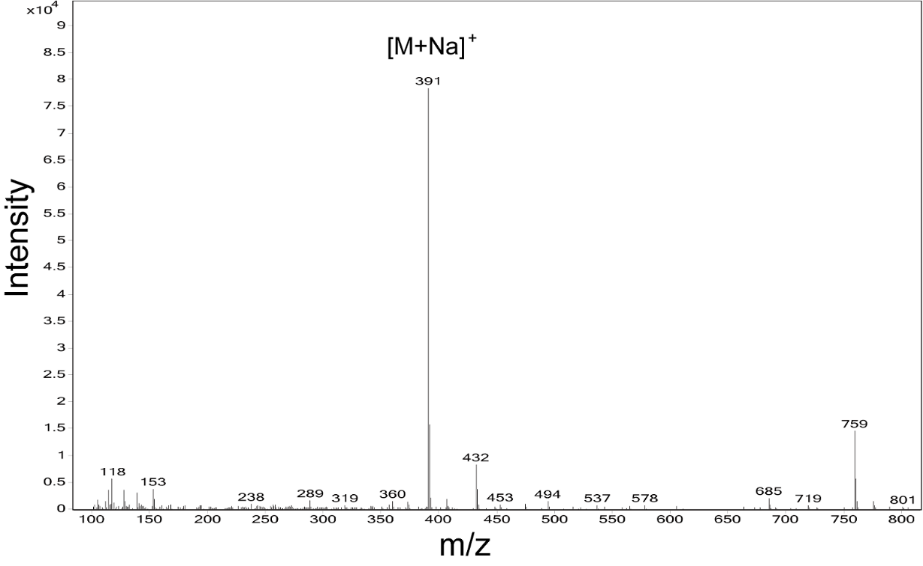


**Fig. S2** MS fragments of DFSK

**Fig. S3** Original western blot images presented in this study

**Fig. S3** (continued) Original western blot images presented in this study

**Supplementary Tables:**

**Table S1.** The equilibrium solubility of DFSK in different solutions ^a^

| **Solvent** | **Equilibrium solubility (μg/mL)** | | |
| --- | --- | --- | --- |
|  | **DFSK** | **ISOF** | **FSK** |
| H_2_O | 786.19 | 144.86 | 22.57 |
| HCl (0.1 mol/L) | 693.79 | 184.84 | 23.39 |
| Phosphate buffer with pH=2.7 | 615.69 | 117.56 | 15.83 |
| Phosphate buffer with pH=3.5 | 610.11 | 116.06 | 14.79 |
| Phosphate buffer with pH=4.5 | 620.50 | 114.52 | 15.31 |
| Phosphate buffer with pH=5.5 | 598.84 | 114.49 | 16.07 |
| Phosphate buffer with pH=6.5 | 585.82 | 111.30 | 15.04 |
| Phosphate buffer with pH=7.5 | 535.58 | 105.74 | 15.20 |

^a^ An excess of DFSK, ISOF, or FSK was weighed and placed into 10 mL test tubes. In each tube, 5 mL of deionized water, 0.1 mol/L hydrochloric acid solution, or phosphate buffer solutions with pH values of 2.7, 3.5, 4.5, 5.5, 6.5, and 7.5 were added. The mixtures were oscillated at 25 °C for 24 hours, then centrifuged at 12,000 r/min for 10 minutes. The supernatant was filtered through a 0.22 μm microporous membrane, and the subsequent filtrate was detected by HPLC. The peak area was measured, and the concentration of each compound was calculated.

**Table S2.** The lipid-water partition coefficient of DFSK in different solutions ^a^

| **Solvent** | **Lipid-water partition coefficient (log P)** | | |
| --- | --- | --- | --- |
|  | **DFSK** | **ISOF** | **FSK** |
| H_2_O | 2.29 | 2.53 | 3.37 |
| Phosphate buffer with pH=3.5 | 2.28 | 2.54 | 3.24 |
| Phosphate buffer with pH=5.5 | 2.31 | 2.62 | 3.27 |
| Phosphate buffer with pH=6.5 | 2.29 | 2.54 | 3.37 |

^a^ Appropriate amounts of DFSK, ISOF, or FSK was dissolved in water-saturated n-octanol to prepare solutions with a mass concentration of 10 mg/mL. Then, 1 mL of each solution was transferred into a 4 mL test tube, and 1 mL of either deionized water or phosphate buffer solution (pH 3.5, 5.5, or 6.5) was added. The mixtures were oscillated at 25 °C for 72 hours, followed by centrifugation at 12,000 r/min for 10 minutes. Subsequently, 20 μL of the aqueous phase was detected by HPLC. The peak area was measured, and the concentration of each compound was determined. The lipid-water partition coefficient was calculated using the formula: log P = log (A / B), where A is the original concentration of the compound in n-octanol, and B is the concentration of the compound in the aqueous phase.
